# Identification and Characterization of a Novel Alpaca Respiratory Coronavirus Most Closely Related to the Human Coronavirus 229E

**DOI:** 10.3390/v4123689

**Published:** 2012-12-12

**Authors:** Beate M. Crossley, Richard E. Mock, Scott A. Callison, Sharon K. Hietala

**Affiliations:** 1 California Animal Health and Food Safety Laboratory System, University of California-Davis, Davis, West Health Sciences Drive, CA 95616, USA; E-Mail: SKHietala@CAHFS.ucdavis.edu; 2 North Carolina Veterinary Diagnostic Laboratory System, Raleigh, NC 27699, USA; E-Mail: Richard.Mock@ncagr.gov; 3 GTCAllison, LLC, Mocksville, NC 27028, USA; E-Mail: SAC@gtcallison.com

**Keywords:** alpaca, human, coronavirus, reverse zoonosis, anthroponosis, respiratory, molecular evolution

## Abstract

In 2007, a novel coronavirus associated with an acute respiratory disease in alpacas (Alpaca Coronavirus, ACoV) was isolated. Full-length genomic sequencing of the ACoV demonstrated the genome to be consistent with other Alphacoronaviruses. A putative additional open-reading frame was identified between the nucleocapsid gene and 3'UTR. The ACoV was genetically most similar to the common human coronavirus (HCoV) 229E with 92.2% nucleotide identity over the entire genome. A comparison of spike gene sequences from ACoV and from HCoV-229E isolates recovered over a span of five decades showed the ACoV to be most similar to viruses isolated in the 1960’s to early 1980’s. The true origin of the ACoV is unknown, however a common ancestor between the ACoV and HCoV-229E appears to have existed prior to the 1960’s, suggesting virus transmission, either as a zoonosis or anthroponosis, has occurred between alpacas and humans.

## 1. Introduction

The isolation of a novel coronavirus associated with an acute respiratory disease in alpacas (Alpaca Coronavirus, ACoV) has previously been described [1]. The virus was initially characterized as a Alphacoronavirus (CoV) based on gene sequencing of a small conserved region of the polymerase gene in the CoV genome. Sequence analysis phylogenetically grouped the alpaca respiratory CoV with several important animal and human coronaviruses, including transmissible gastroenteritis virus and porcine epidemic diarrhea virus of swine, feline coronavirus (FeCoV), canine coronavirus, ferret enteric virus and two common causes of upper respiratory disease in humans, HCoV-229E and HCoV-NL63. The genotype classification additionally differentiated the respiratory alpaca isolate from the CoV previously associated with New World camelids, a Betacoronavirus causing diarrhea and gastrointestinal disease [2]. 

While coronaviruses have been well documented for decades as important respiratory and enteric pathogens of avian [3] and mammalian species [4], and coronavirus-like particles have additionally been described in association with reptiles [5], there has been a notable increase in the number of new coronaviruses detected in recent years, with more than a dozen reported since the zoonotic SARS virus, a Betacoronavirus, was identified in 2003 [6,7]. The apparent emergence of new coronaviruses is believed, in part, to be associated with their genetic mutability and resultant adaptability to new host species [8] combined with better diagnostic tools. 

Among the RNA viruses, the coronaviruses possess the largest genomes, ranging from 26.4 to 31.7 kb. They are also among the most prone to homologous RNA recombination due to their unique use of random template switching during RNA replication, as well as the innate infidelity of RNA replication [6,7]. These virus characteristics combine to generate significant diversity within genotypes of single CoV species, as well as allow coronaviruses to jump host-species and adapt to new ecological niches [7,9,10]. 

While Alphacoronavirus transmission tends to be species-specific, experimental exposures have demonstrated that viral replication and seroconversion can occur across species [11,12,13,14,15], theoretically providing evidence of the first step for adaptation to and emergence as a pathogen in a new host species. 

The extent and role of the novel CoV in alpaca respiratory disease is not well understood, though strong epidemiologic associations have been made between the virus and the emergence of an acute respiratory syndrome in alpacas [1]. The purpose of this study was to complete genetic characterization of the novel alpaca respiratory CoV, through full genome sequencing and phylogenetic analysis, in order to better understand the emergence, possible origins and evolution of the newly detected virus. 

## 2. Results and Discussion

### 2.1. Characteristics of ACoV Genome

**Figure 1:**
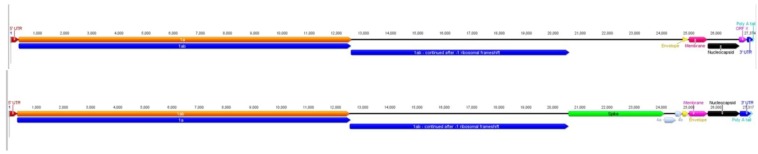
Schematic representation of a comparison between ACoV (top) and the HCoV‑229E (bottom) genome. The figure is drawn to scale; open reading frames are indicated.

The ACoV genome (GenBank Accn. JQ410000) is comparable to other coronaviruses in genome length (ACoV consists of 27,362 bases), the presence of a poly-A tail and an overall 38.5% G+C content (coronaviruses range between 37%–42% G+C [16]). The genome organization consists of six to sevenmajor ORFs and includes short untranslated regions (UTR) at both termini (Figure 1). The major genes are ordered 5'-replicase polyprotein (ORF1a and ORF1ab), spike gene, envelope gene, membrane gene and nucleocapsid gene. As previously reported for HCoV-229E, the sequence UUUAAAC, was also present within the ACoV genome (nt 12,526–12,532) [17]. This sequence is the putative region for a ribosomal frame shift, which creates ORF 1ab. Regions corresponding to previously described ORFs between the spike and envelope genes of other coronaviruses were present, however the ORFs were truncated and/or disrupted. A putative additional open-reading frame found between the nucleocapsid and 3'UTR, designated ORF “X”, was partially created by a 146 nucleotide insertion not present in HCoV-229E (Figure 1). A query of the ORF “X” sequence within the NCBI nucleotide database using a BLAST analysis [18] returned similarities corresponding to proteins found in *Anopheles gambiae*, a malaria-transmitting mosquito (34.3% similarity), and *Candida tropicalis*, a yeast present in the normal human flora (29.0% similarity). Most accessory ORFs do not show homology to other viral or cellular sequences when queried in public databases. Their origin is unknown, and accessory ORFs do not seem to be essential for viral replication in tissue culture [10].

### 2.2. Full-Length Genome Sequence Comparison with Other Coronaviruses

**Figure 2:**
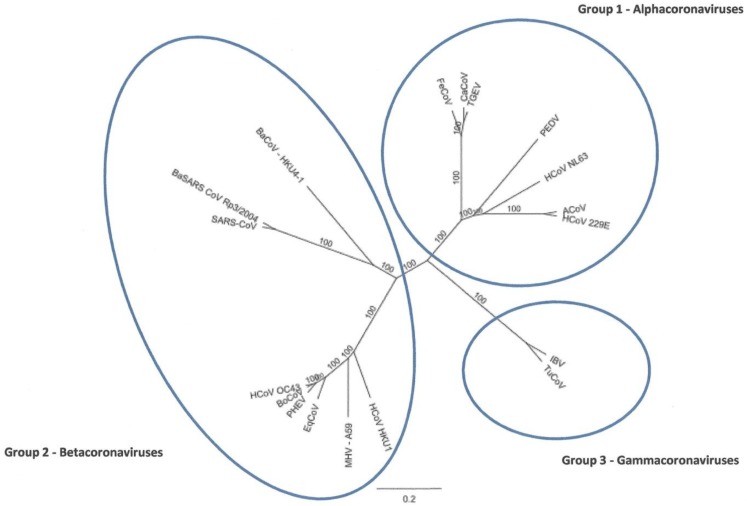
Phylogenetic tree analysis of full-length coronavirus genomes. Alignments were performed using Clustal W and the tree was created using a Neighbor Joining method with no out group (Geneious Pro 5.0.3).

Nucleotide similarity analysis of the ACoV full-length genome clearly placed the virus within the Alphacoronavirus (Figure 2). Within this group, the ACoV was most similar to HCoV-229E at the nucleotide level (92.2%) and less than 70% similar to HCoV NL63. Also, the ACoV had less than 50% genome similarity when compared to the previously reported coronavirus [2] associated with severe diarrhea in alpacas (GenBank Accn. DQ915164). 

Additional characteristics common to the ACoV and other alphacoronaviruses include the length of the genome, gene organization, a potential small ORF downstream of the nucleocapsid gene and an transcription-regulating sequence (TRS) with a core sequence (CS) sequence (AACUAAA) that was found throughout the genome [19]. For example, TRS was found upstream of the spike, membrane and truncated ORF4a genes. The same sequence was also found overlapping the beginning of the ORF “X” gene, while the sequence AUCUAAA was found upstream of the nucleocapsid gene. This transcription-regulating sequence with a core sequence coupled with the lack of a hemagglutinin/esterase (HE) gene clearly supports the inclusion of ACoV into the alphacoronavirus group.

### 2.3. Comparison of ACoV and HCoV-229E Genes/Proteins

Due to the overall similarity, the ACoV genome was compared with the genes from the previously described HCoV-229E (GenBank Accession no. AF304460) (Figure 1, Table 1). The nucleotide pairwise % identities ranged from 86.7%–94.2% (nucleocapsid = lowest; spike = highest). The amino acid pairwise identities ranged from 87.1%–95.0%, with the nucleocapsid having the lowest and the pol 1ab having the highest identity. 

**Table 1:** Comparison of ACoV and HCoV-229E individual gene similarities.

Gene	Nucleotide (%) ^a^	Amino acid (%) ^b^
Pol 1a	92.5	93.2
Pol 1ab	92.9	95.0
Spike	94.2	94.2
Envelope	87.2	91.0
Membrane	89.0	91.2
Nucleocapsid	86.7	87.1

^a^ Nucleotide similarities represent pairwise % identities calculated using the Clustal W method with the IUB cost matrix. ^b^ Amino acid similarities represent pairwise % identities calculated using the Clustal W method with the BLOSUM cost matrix.

### 2.4. Characteristics of the ACoV Spike Gene/Protein and Comparison with HCoV 229 E Spike Gene/Protein

**Figure 3:**
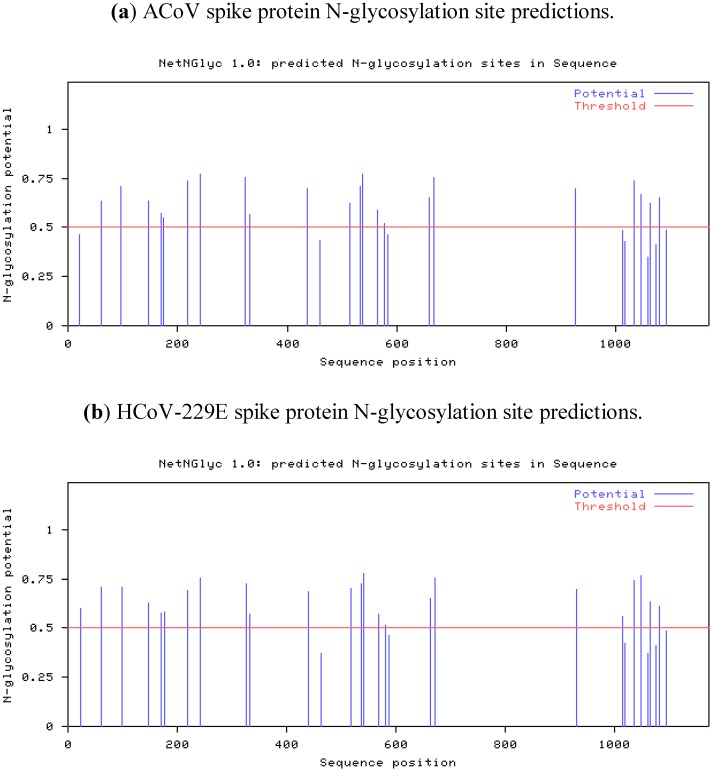
N-glycosylation predictions for the spike proteins of (**a**) the ACoV and (**b**) HCoV-229E. Predictions were done using the NetNGlyc 1.0 server available online [22].

The ACoV spike gene is 3,510 nucleotides in length and is preceded by the common transcription-regulating sequence (TRS) with a core sequence (CS) sequence for Alphacoronaviruses AACUAAA [20]. The spike gene is predicted to encode a protein of 1,170 amino acids, a class I viral fusion protein [21] containing 30 potential N-glycosylation sites, of which only 22 are predicted to be glycosylated (Figure 3) (NetNGlyc 1.0 server [22]). The pattern found in ACoV is essentially identical to the HCoV-229E spike protein. Since HCoV-229E utilizes the Aminopeptidase N protein of their natural host as receptor, a careful assumption is made that ACoV also uses the same receptor mechanism [8]. A basic amino acid cleavage site was not detected within the ACoV sequence, as has been reported for other coronaviruses [23].

**Figure 4:**
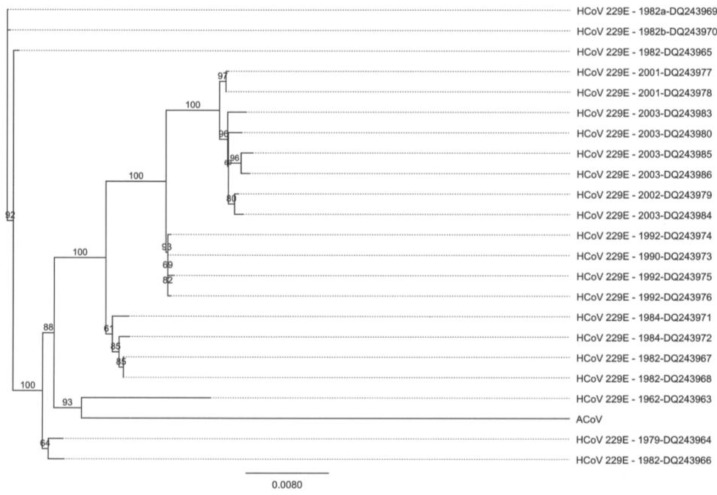
Phylogenetic tree analysis of the nucleocapsid genes from different coronaviruses. Alignments were performed using Clustal W, and the tree was created using a Neighbor Joining method with no outgroup (Geneious Pro 5.0.3).

**Figure 5:**
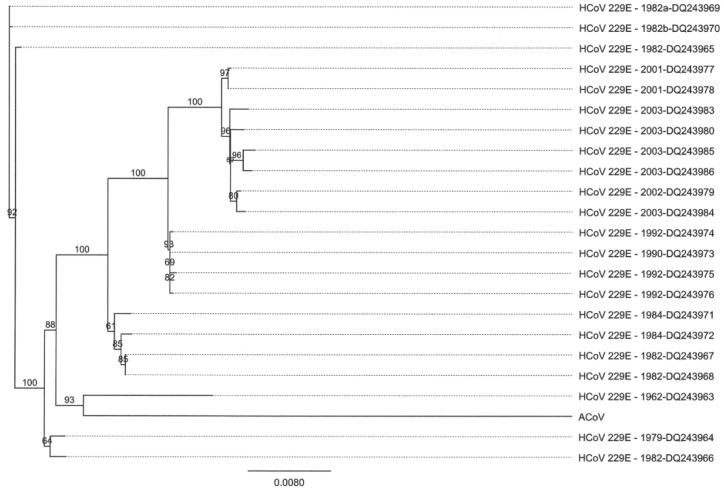
Phylogenetic tree analysis of the spike genes from ACoV and isolates of HCoV‑229E from five decades. Alignments were performed using Clustal W, and the tree was created using a Neighbor Joining method with no out-group (Geneious Pro 5.0.3).

Due to the striking genomic similarity between the ACoV and HCoV-229E, a nucleotide comparison of spike gene sequences from different coronaviruses was performed. Again, the ACoV was most similar to HCoV-229E (Figure 4). Nucleotide comparison of spike gene sequences from the ACoV and isolates of HCoV-229E covering a span of five decades was analyzed, and the ACoV was observed to be most similar to HCoV-229E viruses isolated in the 1960’s to early 1980’s (Figure 5). 

### 2.5. Characteristics of the ACoV Nucleocapsid Gene/Protein and Comparison with HCoV 229 E Nucleocapsid Gene/Protein

The nucleocapsid protein, one of the most abundant viral proteins, is involved in replication processes, assembly and immunity during viral infection [24,25]. The ACoV nucleocapsid gene is 1,158 nt long encoding a protein of 368 amino acids. A 13.3% nucleotide difference resulting in 12.9% amino acid differences were detected between ACoV and HCoV-229E. It is interesting to note that more differences were detected between the nucleocapsid genes of the two viruses than between the spike genes. 

## 3. Experimental Section

### 3.1. Cells and Viruses

ACoV was isolated out of lung tissue of an alpaca with respiratory symptoms using a continuing feline renal cell line. Cytopathogenic effects were detected as viral plaques six days post inoculation in the first passage [1]. Virus was stored at −80 °C until use in small aliquots. 

### 3.2. Viral RNA Preparation

Viral RNA was extracted from cell culture fluid containing the ACoV using the MagMax-96 Viral RNA Isolation kit (Life Technology, Grand Island, NY, USA) or High Pure RNA Isolation kit (Roche, Indianapolis, IN, USA), as described in the manufacturer’s instructions.

### 3.3. RT-PCR Amplification

A two-step reverse transcriptase polymerase chain reaction RT-PCR was used to produce overlapping DNA fragments from the ACoV RNA (Table 1). Reverse transcriptase reactions were performed using the MonsterScript™ 1^st^-Strand cDNA Synthesis kit (Epicentre Biotechnologies, Madison, WI, USA). Briefly, 5 μL of ACoV RNA was mixed with 9 μL of water and 1 μL of ACoV genome specific primer (20 μM) and then incubated at 65 °C for 1 minute, followed by placing the tube on ice for 1 minute. Next, 4 μL of 5× premix was added to the mixture along with 1 μL of RT enzyme. The mixture was incubated at 42 °C for 5 minutes and then 60 °C for 1 hour. The reaction was terminated by incubation at 90 °C for 5 minutes. The cDNA was used immediately as a template for PCR or stored at −20 °C for later use. For PCR, the Failsafe PCR System (Epicentre Biotechnologies, Madison, WI) was used. Briefly, 1 μL of cDNA from a RT reaction was mixed with 9.25 μL of water, 1 μL of ACoV fragment specific forward primer (20 μM) (Supplementary Table 1), 1 μL of ACoV fragment specific reverse primer (20 μM) (Table 1), 0.25 μL of Taq enzyme and 12.5 μL of the predetermined best premix buffer for each reaction. The PCR was performed using the thermocycling protocol of 95 °C for 2 minutes, followed by 35–45 cycles of 95 °C for 30 seconds, 50–60 °C for 30 seconds, 72 °C for 1–3 minutes, followed by a 7 minute elongation step at 72 °C. The specific annealing temperature and elongation times were fragment-specific and varied based on the calculated melting temperature for each primer set and the size of the fragment being amplified. 

### 3.4. 5' RACE and 3' RACE

DNA fragments representing the 5' and 3' ends of the ACoV genome were amplified using the FirstChoice® RLM-RACE kit (Life Technology, Grand Island, NY, USA), per the manufacturer’s recommended procedure. 

### 3.5. Primers

The primer sets used to amplify the ACoV overlapping DNA fragments are listed in the Supplementary Table 1. The specific primer sets were designed to the full-length human coronavirus 229E sequence (GenBank accession # AF304460). Some of the primer sequences contain mismatches when compared to the resultant ACoV genome. The RT-PCR amplification and internal sequencing primers (sequences not shown) were designed using freely available primer selection programs available through Integrated DNA Technologies [26].

### 3.6. TOPO TA Cloning

Some RT-PCR fragments were cloned into pCR® XL-TOPO (Life Technology, Grand Island, NY, USA) following the manufacturer’s recommendations to facilitate DNA sequencing and subsequent genome assembly.

### 3.7. DNA Sequencing and Analysis

The overlapping DNA fragments corresponding to the ACoV full-length genome were sequenced by automated Sanger sequencing (Functional Biosciences, Madison, WI, USA). The genome coverage for the sequencing of the ACoV genome was approximately three-times coverage using Sanger sequencing. All DNA sequence data assembly and phylogenetic analyses were performed with Geneious Pro 5.0.3 (Biomatters, Newark, NJ, USA).

## 4. Conclusions

The novel alpaca coronavirus described here was isolated during an outbreak of severe respiratory disease and associated abortions in alpaca in 2007, following co-mingling of a large number of animals at a national exposition and sale [1]. Subsequent large outbreaks of the disease have not been reported, leaving questions remaining as to the origins of the outbreak virus, as well the persistence of ACoV in the national alpaca population. Similarities between the whole genome sequences of ACoV and HCoV-229E, a coronavirus first isolated around 1960, [27,28] are evident. Though speculative, the similarities suggest a transmission event between humans and alpacas. Molecular studies suggest the possibility that HCoV-229E was acquired by humans from a host switching event a few hundred years ago and most likely involved bats [29]. Therefore, it is not surprising to find bat CoV isolated from *Hipposideros* bat species so closely grouped with the HCoV-229E and ACoV viruses in the phylogenetic analysis of the nucleocapsid gene. Although it is possible that the speculative transmission event between humans and camelids was recent, the evolutionary ancestor appears more closely related to HCoV-229E viruses isolated around 1960, and ACoVs may more realistically have been circulating for decades at low levels in alpaca populations. New world camelids were introduced into the U.S. as recently as 1985, meaning associated health management and diagnostic medicine practices are still relatively new. It is not unreasonable to assume that ACoV could have caused respiratory disease in individual animals and herds, without accurate diagnosis for decades. In the United States, alpacas are generally maintained in small herds where exposure to new infectious agents would occur primarily when unrelated naive and actively shedding animals are co-mingled. This occurred prior during the 2007 outbreak and helps to explain the large number of severely affected animals at a single point in time. 

Most Alphacoronaviruses, including AcoV, utilize Aminopeptidase N of their natural host as a receptor, and host tropism differences of viruses are caused by the ability of their spike proteins to detect small species-specific amino acid differences in the aminopeptidase N [8,13]. Despite the host specificity of this receptor, all of those viruses are known to bind and utilize feline Aminopeptidase N, suggestive of a common coronavirus ancestor infecting feline species [30]. This would explain the ability to grow ACoV on feline origin cell lines, such as Crandell-feline kidney cells (CRFK) [31], and the failure to propagate virus on numerous other mammalian cell lines [1].

Due to the severe clinical signs associated with ACoV, the Camelid industry has expressed interest in the use of vaccination as a possible disease control option. The question was raised if a potential use of the bovine coronavirus vaccine might protect camelid species from the respiratory ACoV. Evaluating the sequence data due to potential binding mechanism of the ACoV as a Alphacoronavirus shows a completely different pattern compared to the enteric alpaca coronavirus and the bovine coronavirus, both belonging in the Betacoronavirus group. Betacoronaviruses are known for using mostly sialic acid binding activity for viral entry [32], whereas alphacoronaviruses can utilize Aminopeptidase N binding as a mechanism. Based on genome sequencing information, it is presumed that vaccines targeting Betacoronaviruses will likely not prevent infection with Alphacoronaviruses, such as AcoV, due to the different cell entry mechanism.

The spike gene has the critical role of virus entry and membrane fusion processes in the host cell, however, work with chimeric viruses also shows the spike protein can influence tropism and pathogenesis of certain coronaviruses [33,34,35]. It is interesting to note that the spike genes, which tend to contain hypervariable regions, for the ACoV and HCoV-229E are more similar to each other than the nucleocapsid genes. Evolutionary studies monitoring HCoV-229E found only limited variation within the S gene nucleotide sequences in contrast to the evolutionary pattern of spike genes in other human coronaviruses [36,37], suggesting the spike protein is evolutionarily stabile. Nucleocapsid sequence comparisons among feline coronavirus isolates suggest the mutation patterns can be associated with geographic origin, rather than with virulence patterns [38]. It would be interesting to compare nucleocapsid sequence of South American HCoV-229E strains to the ACoV nucleocapsid strain.

The ACoV described in this sequence analysis has several unique characteristics. The virus has a truncated or disrupted ORF 4a/4b, which has been reported for other, but not all HCoV-229E isolates [39]. Whether this ORF truncation is real or a laboratory artifact (*in vitro* growth in cell culture) remains to be elucidated. Also, as compared to HCoV-229E, the ACoV has an extra putative ORF “X” overlapping the 3' end of the nucleocapsid gene. Further laboratory experiments will be required to determine if this gene is expressed and, if so, the protein’s *in vivo* function. 

It is critical to characterize and understand new and emerging viruses, particularly in the context of zoonosis, whether transmitted from animal to man or man to animal. Coronaviruses, considered the most frequent source of the common cold in humans, are also widely dispersed throughout mammalian and avian populations. They have shown an ability to jump species, resulting in mild to serious zoonotic disease threats. The current ACoV genomic data provides strong evidence of an evolutionary relationship between HCoV-229E and AcoV. Whether the virus represents a recent cross-species transmission or a more distant transmission between humans and alpacas is yet to be determined. It would be interesting to analyze South American HCoV-229E isolates that were obtained in different years to determine the genetic similarities to the ACoV. This line of investigation might shed more light on the possibility of human to alpaca or alpaca to human transmission events and help to calculate when such an event might have occurred. 
